# Topical tacrolimus 0.1% ointment for treatment of cutaneous Crohn’s Disease

**DOI:** 10.1186/1756-0500-6-19

**Published:** 2013-01-18

**Authors:** Shantini A Rice, Pick N Woo, Emad El-Omar, Ronald A Keenan, Anthony D Ormerod

**Affiliations:** 1 Department of Dermatology, Aberdeen Royal Infirmary, Foresterhill, Aberdeen AB25 2ZB, UK; 2 Division of Applied Medicine, University of Aberdeen, Foresterhill, Aberdeen AB25 2ZB, UK; 3 Department of Surgery, Aberdeen Royal Infirmary, Foresterhill, Aberdeen AB25 2ZB, UK

## Abstract

**Background:**

Cutaneous Crohn’s Disease is a notoriously difficult condition to treat and causes significant morbidity, impacting heavily on quality of life. This is the first study in adults examining the effect of topical tacrolimus on the different cutaneous manifestations of Crohn’s Disease.

**Methods:**

This open label observational study of 20 patients with heterogeneous forms of cutaneous Crohn’s disease used topical tacrolimus 0.1% ointment once daily to affected areas for 12 weeks with a maximal total dose of 90g. Therapy was stopped at 12 weeks to assess whether the condition relapsed. Thereafter relapsing patients optionally continued an open label extension of topical tacrolimus therapy and were observed for a total of 12 months.

**Results:**

Of seventeen patients completing the twelve-week study, fifteen improved using a specifically designed physicians’ global severity scale. One patient cleared, four showed a pronounced improvement (51-75%) and ten demonstrated a mild (1-25%) or moderate improvement (25-50%) in twelve weeks. Over twelve months eleven patients remained in the study, nine of which improved, one cleared and one showed no change. Perineal disease responded better with two out of twelve clearing, four showing pronounced benefit and four mild to moderate improvement. Long-term application of 0.1% tacrolimus applied to broken skin and mucosa was safe and serum levels of tacrolimus were undetectable in all subjects throughout the study.

**Conclusion:**

0.1% tacrolimus ointment was safe and effective in treating cutaneous manifestations of Crohn’s disease, particularly perineal disease and pyoderma gangrenosum, yet it seldom cleared the condition.

**Clinical trial registration:**

ClinicalTrials.gov Protocol Registration System ID: 33000332

## Background

Crohn’s disease is a chronic inflammatory bowel disease of unknown aetiology with distinctive mucocutaneous manifestations. Cutaneous Crohn’s Disease (Cutaneous CD) is relatively common with an incidence between 11% and 44% of individuals with Crohn’s disease of the bowel being affected. The Grampian region in Scotland appears to have the highest incidence of cutaneous Crohn’s in Europe [1-5]. It causes significant morbidity and severely impacts upon quality of life [6,7].

There are three distinct manifestations of cutaneous Crohn’s [8-10]. The most common types are perianal disease (PD), often with fissuring or fistulae, granulomatous cheilitis and peristomal pyoderma gangrenosum (PPG). The latter is often related to the severity of the gastrointestinal disease and has been treated with topical tacrolimus [11].

The second category encompasses conditions that have persistently been reported as having a strong association with Crohn’s disease, such as classical pyoderma gangrenosum, erythema nodosum, erythema multiforme, acrodermatitis enteropathica and epidermolysis bullosa acquisita.

The last and most uncommon type is that of granulomatous cutaneous lesions which are non-contiguous with the gastro-intestinal tract. This is often referred to as metastatic Crohn’s Disease (MCD) and has a predilection for skin folds but may also involve other sites such as the face, vulva, penis, trunk and limbs [12].

Severe perineal disease, PPG and MCD are notoriously difficult to treat and are frequently resistant to corticosteroids, oral antibiotics, dapsone, thalidomide and immunomodulating agents such as azathioprine, ciclosporin and sulphasalazine. Activation of Th1 lymphocytes plays a crucial role in the mucosal destruction and this can be inhibited by the calcineurin inhibitors ciclosporin A and tacrolimus [13]. As tacrolimus is 10–100 times more potent than ciclosporin [14] and better able to penetrate skin well due to its lower molecular weight we hypothesise that tacrolimus 0.1% ointment will be an effective and safe treatment in the management of cutaneous Crohn’s Disease.

Tacrolimus has become an important addition to therapy for atopic dermatitis [15] and other inflammatory dermatoses including psoriasis, allergic contact dermatitis, graft-versus-host disease and recalcitrant leg ulcers with rheumatoid arthritis, all with beneficial outcome [16-21]. Of particular note, it has been reported to be effective in erosive oral lichen planus [22,23] and in otherwise treatment-resistant pyoderma gangrenosum [11].

There are few studies that evaluate the benefit of topical tacrolimus on cutaneous Crohn’s. A series of subjects with oral and perianal Crohn’s disease reported efficacy with marked improvement in 7 out of 8 children of 0.05% tacrolimus made up from the intravenous formulation [24]. A more recent study randomised 19 patients to 0.1% topical tacrolimus with 3 of 4 patients healing ulcerating disease but poor responses for fistulae [25]. There are case reports of improvement of parastomal pyoderma gangrenosum [11] with topical therapy. Increasingly tacrolimus has been shown to be useful as a systemic agent in Crohn’s disease [26-29] and in treating resistant disease [27,30] particularly showing efficacy for fistulating disease [31,32].

This study is the first to look at the effect of topical tacrolimus 0.1% on the different cutaneous manifestations of Crohn’s in adults. We found it to be effective in reducing the severity of several of the cutaneous manifestations of Crohn’s disease particularly pyoderma gangrenosum and perianal disease. However the disease was seldom cleared, improvement was slow and rapid relapses were seen where treatment was not maintained. Concerns of possible systemic absorption of tacrolimus [33] when applied to broken skin and mucosal surfaces were addressed in this study.

## Methods

This was an open label study of 20 patients with cutaneous Crohn’s Disease using topical tacrolimus 0.1% ointment once daily to affected areas for 12 weeks with a maximal total dose of 90g. Therapy was stopped at 12 weeks to assess whether the condition relapsed. Thereafter relapsing patients optionally continued an open label extension of topical tacrolimus 0.1% and were observed for a total of 12 months. The study was conducted at the Dermatology Department of Aberdeen Royal Infirmary. The protocol was approved by North of Scotland Research Ethics Committee and all patients provided written informed consent to participation and to publication of photography and clinical databefore inclusion in the study.

We recruited patients by distribution of information leaflets to consultant gastroenterologists and surgeons in Aberdeen Royal Infirmary between October 2002 and December 2003. Patients suitable for the study were offered an appointment within two weeks of contacting the department.

Patients aged twelve years or more with a willingness and capability to follow the study procedure were considered for inclusion. Each subject had to have a confirmed diagnosis of Crohn’s disease of at least three months’ duration confirmed by radiography, endoscopy or pathological examination and were required to have a skin manifestation of Crohn’s disease. Both male and female subjects with reproductive potential required to be on an acceptable form of birth control for the duration of the study. Long-standing, concomitant immunosuppressive therapy was allowed if the dose was stable and not controlling the skin problem.

Patients were excluded if they had had a known sensitivity to tacrolimus, a change in aminosalicylate dosage in the four weeks prior to screening, were on oral steroids at over 40 mg per day, had been commenced on methotrexate, azathioprine or ciclosporin within the last two months or an anti- TNF monoclonal antibody within the three months prior to screening. Patients having had a stoma fashioned less than three months before enrolment, those with an immunocompromising disease, a diagnosis of malignancy within the last five years, any other condition, past or present treatment thought by the investigator to render the subject ineligible for the study were excluded, as were those with erythema nodosum.

Subjects were instructed to apply topical tacrolimus 0.1% ointment to their skin lesions once daily for three months. Therapy was then stopped for a month and if relapse occurred subjects could recommence therapy for up to 1 year.

The primary outcome measure was assessment of standardised digital photography by three independent assessors. As no validated measures were available the investigators specifically designed a Physicians’ Global Severity Scale to assess global cutaneous disease severity when patients had received at least 12 weeks’ treatment. The Scale was used to assess improvement by examination of photographs taken before and during treatment (Table 1).

**Table 1:** Physicians global severity scale

	
6 Worse	
5 No change	
4 Mild improvement: 1-25%	
3 Moderate improvement: 26-50%	
2 Pronounced improvement: 51-75%	
1 Almost clear: 76 to <100% improvement	
0 Completely clear: 100% improvement	

Other methods used for assessment over 52 weeks included a global self-assessment score and the validated Perineal Disease Activity Index (PDAI), where appropriate for perineal disease. A potential confounder in this study was a deterioration of cutaneous signs secondary to a systemic worsening of Crohn’s disease. The Crohn’s Disease Activity Index (CDAI) was used to control for this.

The PDAI incorporates five elements: the presence or absence of discharge, pain or restriction of daily living, restriction of sexual activity, the type of perianal disease, the degree of induration, yielding a composite score ranging from 0 to 20 with higher scores indicating more severe disease. The CDAI is a validated test which incorporates eight related variables: the number of liquid or very soft stools per day, the severity of abdominal pain or cramping, general well-being, the presence or absence of an abdominal mass, the use or non-use of anti-diarrhoeal drugs, the haematocrit and body weight. The scores range from 0 to 600 with scores below 150 indicating remission and those above 450 indicating severe illness.

Safety evaluations included recording of side effects, physical examination including tracing and photographs, and vital signs measured by a single investigator during the study. As the tacrolimus was being applied to areas lacking a skin barrier, fistulae, ulcerated and mucosal surfaces, systemic tacrolimus levels were measured at every visit to assess any significant systemic absorption. Blood pressure, urinalysis and renal function were measured and remained constant throughout the study.

## Results and discussion

Twenty patients aged between 12 and 66 years with 12 males and 8 females were enrolled in the trial. Nine patients had perianal disease; five granulomatous cheilitis; one metastatic Crohn’s disease; two pyoderma gangrenosum, one in the peristomal region. The 3 remaining patients had perianal disease plus either PG or MCD.

**Figure 1:**
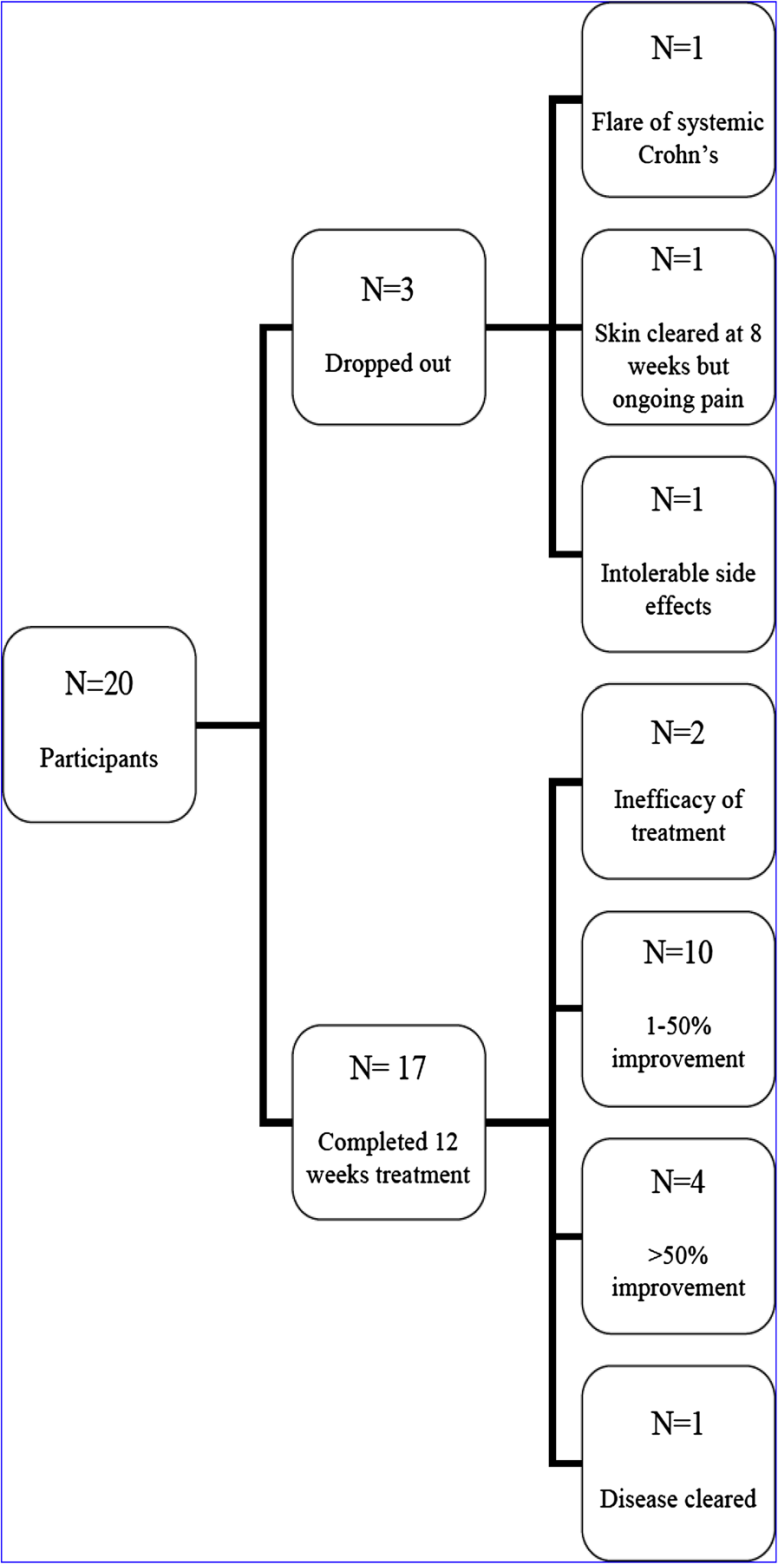
Patient flowchart.

Out of the twenty patients, seventeen completed 12 weeks of treatment. The primary outcome measured using the physicians’ global severity scale from clinical photographs demonstrated a median improvement of “moderate improvement” (4 on the 6 point scale) equivalent to 26-50% improvement in the seventeen patients. The three independent assessors showed a fair intra-observer agreement with weighted Kappa-scores of 0.31, 0.38 and 0.38 (0.2-0.4 = fair agreement) (Figure 1).

**Figure 2:**
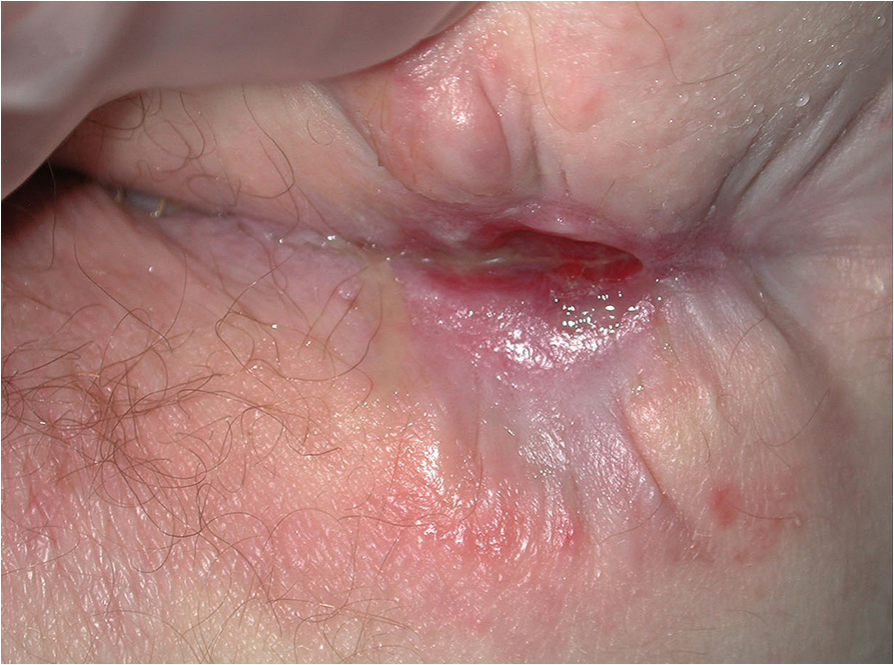
Patient 19 with perineal disease.

**Figure 3:**
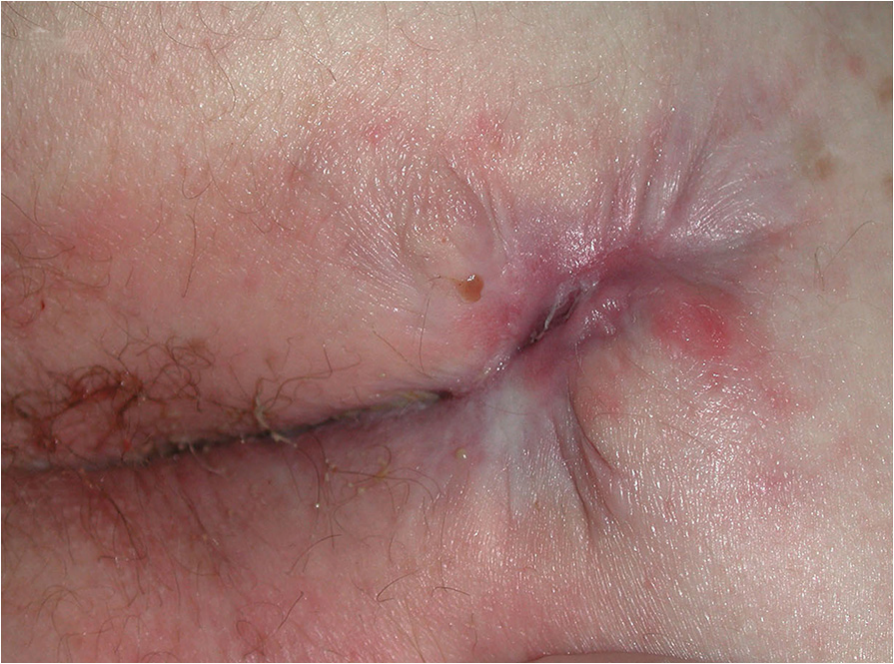
Patient 19 with perineal disease: cleared with course of treatment.

**Figure 4:**
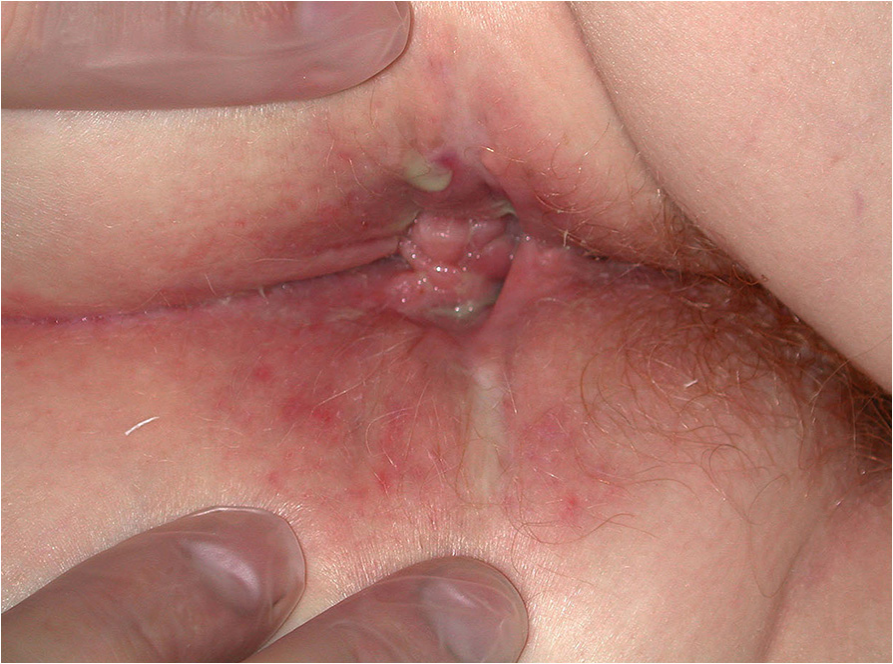
Patient 13 with perineal disease.

**Figure 5:**
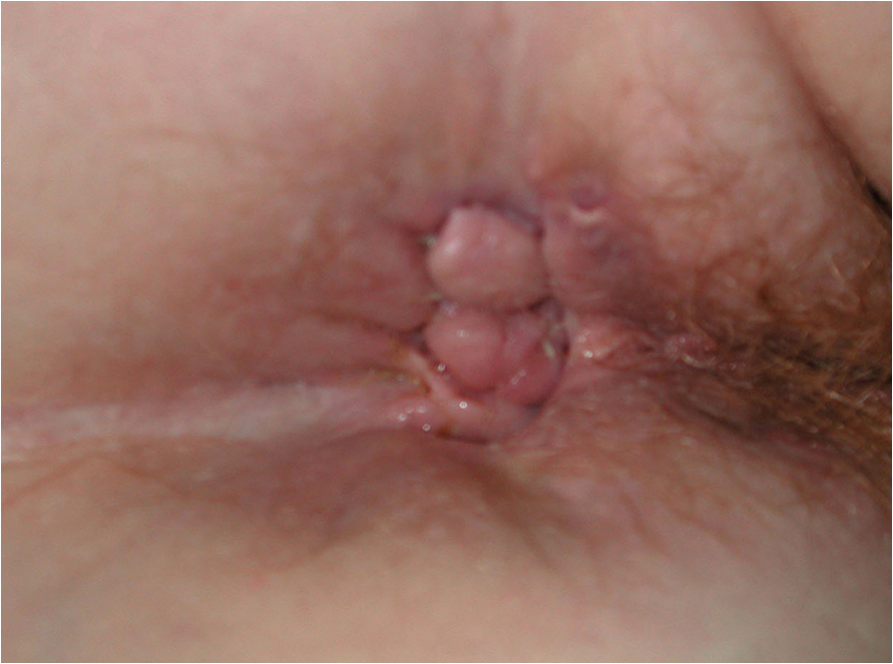
Patient 13 with perineal disease: moderate improvement with treatment.

At 12 weeks, the greatest improvement with treatment was noted in patients with PD. Disease clearance was noted for one patient (Figures 2 and 3). Another patient whose PD cleared rapidly left the trial early no longer requiring treatment. Pronounced improvement was evident in four patients, two with PD and two with PD and MCD, (although with a moderate response of the MCD to treatment in one). Four patients had a mild or moderate improvement (see Figures 4 and 5). A patient with perianal disease and sinus formation did not improve at this point.

There were three patients with PG and two had mild or moderate improvement; the other withdrew at 4 weeks due to intolerable side effects but had improved up to this point.

**Figure 6:**
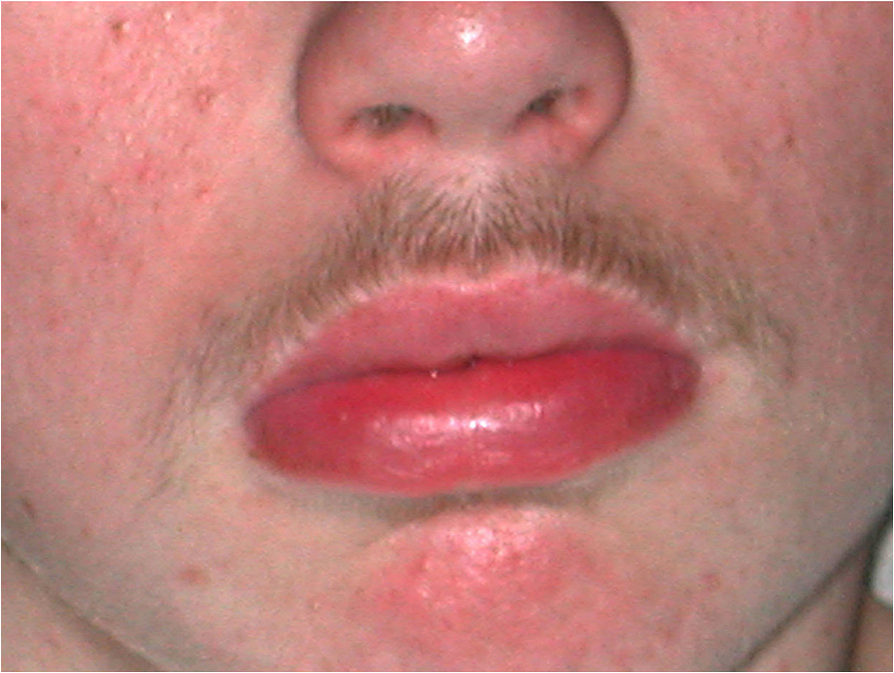
Patient 18 with granulomatous cheilitis.

**Figure 7:**
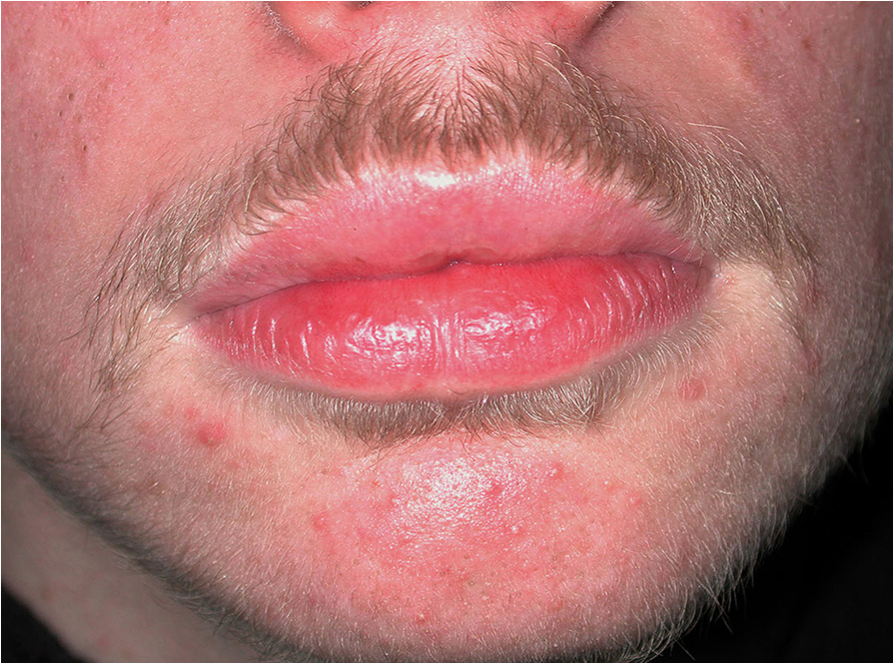
Patient 18 with granulomatous cheilitis: moderate improvement of lip swelling with treatment.

The five patients with granulomatous cheilitis all improved but only to a mild or moderate extent (Figures 6 and 7) and four relapsed after stopping therapy. The patient with MCD did not experience any improvement in disease at 12 weeks.

Ten patients relapsed within 3–9 days of cessation of treatment. Eleven patients chose to continue the study for a further 36 weeks. One of these patients (with PD) had disease clearance as measured by the Physicians’ Global Severity Scale, 9 had an overall improvement in their cutaneous Crohn’s disease (6 with PD, 1 with GC, 2 with PG) and 1 showed no change (PD). Interestingly, the two patients with PG had shown only a mild or moderate improvement at week 16, but had pronounced improvements by the end of the 52 weeks (Table 2).

**Table 2:** Patient demographics and overall response to treatment

	**AGE years**	**SEX**	**CONDITION**	DURATION years*	**CONCOMITANT THERAPIES**	**OVERALL RESPONSE TO TREATMENT ^**	** NOTES**
1	35	F	PD	1.5	Azathioprine 1yr, prednisolone	Cleared	Relapsed on stopping at 12 weeks
2	27	M	PD, MCD	4	Azathioprine 1yr, Prednisolone, Asacol	Two areas healed rest improved prior to exiting	Dropped out due to flare of Crohn’s
3	60	F	PG	33	Azathioprine 2yr, Predfoam, Asacol	Improved	Dropped out - intolerable pain from ointment
4	65	F	PD	10	Nil	Improved	Relapsed on stopping at 12 weeks
5	33	M	GC	12	Nil	Improved initially, worsened during extension	Relapsed on stopping at 12 weeks
6	37	F	GC	5	Prednisolone	Improved	Less frequent and severe swelling.
7	20	M	PD	3	Azathioprine 5yr, Methotrexate	Improved	Dropped out - severe flare of systemic disease
8	12	M	GC	5	Nil	Moderate improvement	Relapsed on stopping at 12 weeks
9	32	M	MCD	4	Azathioprine 2yr	No change	
10	66	M	PD	1.5	Nil	Initial improvement	Relapsed on stopping at 12 weeks
11	42	M	PD	17	Nil	Improved	
12	16	M	GC	13	Nil	Mild improvement	Relapsed on stopping at 12 weeks
13	56	F	PD	15	Asacol, Predfoam	Moderate improvement	Relapsed on stopping at 12 weeks
14	29	F	PD	1.5	Nil	No change	
15	35	M	PD	0.75	Nil	Pronounced improvement	Relapsed on stopping at 12 weeks
16	63	F	PG, PD	6	Nil	Pronounced improvement	Systemic disease flared week 9
17	33	M	PD	5	Nil	Skin healed at 8 weeks but pain continued	Exited trial at week 8
18	15	M	GC	10	Nil	Moderate improvement of lip swelling	Fewer mouth ulcers, relapsed
19	30	M	PD, MCD	25	Nil	Cleared	
20	38	F	PPG	1.5	Prednisolone, methotrexate, infliximab	Pronounced improvement	Relapsed on stopping at 12 weeks

* Duration of diagnosis of cutaneous Crohn’s disease; ^ Physicians Global Assessment; *PD* perineal disease; *GC* granulomatous chelitis; *MCD* mixed connective tissue disease; *PPG* peristomal pyoderma gangrenosum.

**Figure 8:**
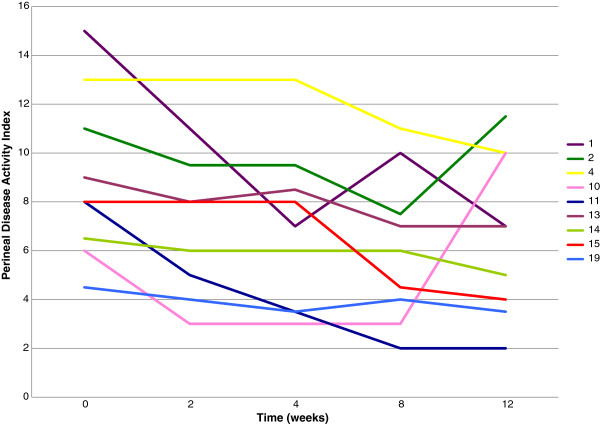
**Patients with Perineal Disease who completed 12 weeks of treatment showing change in Perineal Disease Activity Index during this period. **Note Patient 2 had a flare of Crohn’s unrelated to treatment; Patient 10 flared after stopping treatment.

As perineal disease was the most frequent cutaneous manifestation, a subgroup analysis of patients with PD was conducted. Three of these patients did not complete 12 weeks of treatment; one dropped out due to a systemic flare at 9 weeks but had pronounced improvement in perineal disease at the point of exit and another patient exited early when the perineal disease cleared. The remaining patient with exiting early had a flare in systemic Crohn’s. Of those completing 12 weeks of treatment, disease clearance was noted for one patient, pronounced improvement in three and a further four had a mild or moderate improvement (Figure 8).

**Figure 9:**
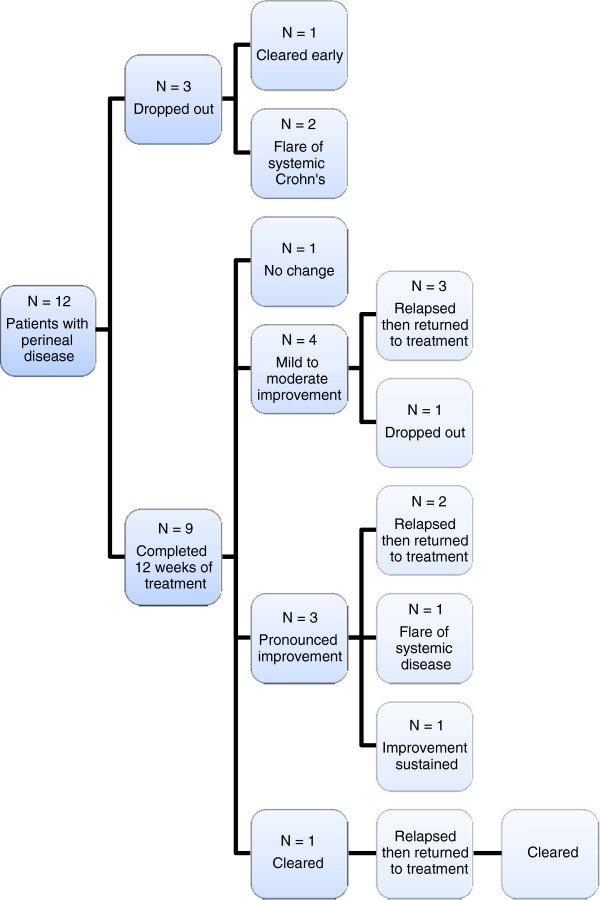
Flowchart of patients in study with Perineal Disease showing responses to initial 12-week treatment course then open-label extension.

However, at week 12 when treatment was stopped, six of these improvers relapsed and elected to join the trial extension. With continued treatment beyond the initial course, the improvement was recovered and a further patient achieved clearance of PD. Only one patient could maintain clinical improvement with cessation of treatment at week 12 (Figure 9).

Eight patients reported burning, itching or discomfort with topical application of tacrolimus. Four patients described this side effect as a brief symptom immediately after application; another dropped out as a result of it. Conversely, five patients commented on improvement of local discomfort with continued use of topical tacrolimus. Systemic absorption of tacrolimus was undetectable in all patients throughout the study with blood levels of tacrolimus at every visit being below the limits of detection despite daily application to mucosal surfaces and broken or ulcerated skin in all cases.

## Conclusion

There is limited evidence for the efficacy of the commercial preparation of 0.1% tacrolimus (Protopic®, Fujisawa) in the treatment of perineal Crohn’s disease and pyoderma gangrenosum. We found it to be effective in reducing the severity of several of the cutaneous manifestations of Crohn’s disease.

However, our study found that it seldom cleared cutaneous Crohn’s disease. Improvement was slow and rapid relapses were seen when treatment was not maintained; ten patients relapsed within 3 to 9 days of stopping treatment at week 12 and thus treatment was extended to 12 months. The problem of relapse after abrupt cessation of treatment was noted to occur in two of the patients in the study conducted by Casson et al [24].

The most favourable results were seen in our patients with perianal disease and pyoderma gangrenosum with reduction of inflammation and promotion of ulcer healing. In the case of PD, treatment needed to be continued in most cases in order to maintain the response. PG, was slower to respond and seemed to reach a peak improvement well beyond 12 weeks where treatment was continued. Indeed topical tacrolimus at lower concentrations has previously been shown to be useful in the management of perineal Crohn’s disease and peristomal pyoderma gangrenosum in children [24]. However, we found that fistulae and sinuses tended not heal, in keeping with the findings of Hart *et al*[25].

Patients with granulomatous cheilitis showed mild improvement with reduced severity and frequency of flares. Associated aphthous ulceration in our patients also improved, a tendency noted by Casson *et al.* who found improvement in 3 children with oral disease [24].

The efficacy of topical immunosuppressive agents can be limited by the size of the molecule in relation to the skin barrier. However, the predominantly ulcerative lesions of cutaneous Crohn’s disease are without an epidermal barrier and thus more permeable, maximising the therapeutic potential of a topical drug. Conversely, skin absorption is impeded in fistulae by their anatomy and flow of exudate. Systemic administration is however, effective for fistulae [31].

Future work in this field should investigate the efficacy of a long-term maintenance regime of topical tacrolimus in preventing relapses of cutaneous Crohn’s, similar to that used in the treatment of atopic eczema.

### Limitations of this study

Our study was necessarily limited by the small number of participants meeting the inclusion criteria who wished to participate. We acknowledge that the separate cutaneous features of Crohn’s disease are heterogeneous but in the context that they present clinically and with very limited published evidence, such an observational prospective study of a new therapy adds to our knowledge of treatment. We also had to develop our non validated rating scale to accommodate the heterogeneous manifestations although supplementing validated scores including PDAI for relevant cases. Although erythema nodosum (EN) is the commonest cutaneous manifestation of Crohn’s disease, it was not included in this study. As the pathology found in EN is within the subcutaneous fat with an intact overlying epidermis, it is unlikely that topical tacrolimus could penetrate to or be effective at this level of the skin. Additionally, EN tends to be self-limiting.

The primary outcome measure was dependant on the quality of both the photographers’ skill and the prints and the Kappa score reflected only fair agreement between the assessors. Patients with enteral Crohn’s disease are often on some long-term preventive steroid or immunomodulatory therapy to control their enteral disease. Seven of our patients were taking low dose prednisolone or azathioprine, but these long term maintenance therapies were ongoing for at least a year at doses stable for the preceding three months, and were not sufficient to control the cutaneous disease. Where a flare in systemic disease led to new immunosuppressives being prescribed or high dose steroids patients were discontinued from the study. As this was an open label trial without a control arm, we cannot report on the natural history of untreated lesions of cutaneous Crohn’s disease although placebo treatment in the study by Hart [25] did not lead to improvement despite concomitant systemic immunosuppressive therapies.

## Competing interests

ADO received an honorarium in 2007 from Astellas.

## Authors’ contributions

All authors read and approved the final manuscript. PW, SR, EE-O, RAK, ADO had full access to all of the data in the study and take responsibility for the integrity of the data and the accuracy of the data analysis. Study concept and design: PW, EE-O. RAK, ADO. Acquisition of data: PW, EE-O. RAK, ADO. Analysis and interpretation of data: PW, SAR, ADO. Drafting of the manuscript: SAR, ADO. Critical revision of the manuscript for important intellectual content: ADO. Statistical analysis: SAR, ADO. Obtained funding: ADO, PW.

### Funding

This study was supported in part by Fujisawa now Astellas. The sponsors had no role in the design and conduct of the study, in the collection, analysis, and interpretation of data or in the preparation, review, or approval of the manuscript.
